# The effect of combining laser and nanohydroxy-apatite on the surface properties of enamel with initial defects

**DOI:** 10.4317/jced.54371

**Published:** 2018-05-01

**Authors:** Dina-Wahied El Assal, Ali-Mohamed Saafan, Dina-Hassan Moustafa, Marwa-Adel Al-Sayed

**Affiliations:** 1 Professor of Dental Laser Applications National Institute of Laser Enhanced Sciences,Cairo University; 2 Professor of Dental Biomaterials Materials Department, Cairo University; 3 Assistant Professor of Inorganic Chemistry Department, National Research Center Giza, Egypt

## Abstract

**Background:**

The aim of this study was to evaluate the effect of combining fractional CO2 LASER and nanohydroxy apatite on surface microhardness and color of enamel with initial defects.

**Material and Methods:**

Two types of nano hydroxylapatite (nHAP) was prepared; Pure hydroxyapatite (nHA) and Fluoro hydroxyapatite (nFHA), Sixty extracted premolar teeth without visible caries or structural defects on enamel surface were used, immersed in 10 ml of a demineralizing solution for 2 weeks to create artificial white spot lesions, they were randomly allocated into two groups; Group 1: nHA, Group 2: nFHA, each group is then subdivided into 2 subgroups (A and B) where two different *in vitro*remineralization procedures have been performed, the first procedure utilizes a 10 wt% nHA aqueous slurries only, the second was first exposed to irradiation from a fractional CO2 laser then (nHAP) was applied. Microhardness and color were measured using a micro-Vickers hardness tester and spectrophotometer respectively.

**Results:**

Laser treated teeth in both groups showed the highest mean hardness and lowest color difference where ΔE was less than 3.3 units, in both tests the pure type of nanohydroxyapatite gave better results than the nanofluroapatite type.

**Conclusions:**

Nano-hydroxyapatite has remarkable remineralizing effects on initial lesions of enamel, certainly higher when combined with laser application.

** Key words:**CO2 LASER, Enamel remineralization, Nanohydroxy apatite.

## Introduction

The phenomenon of reversal of incipient or early enamel defects forms an important part of prevention since it is a dynamic process with interspersed periods of demineralization and remineralization leading to apparent repair of the lesion (1). Some products are designed to mimic the process of remineralization for the purpose of prevention and therapeutic strategies for early enamel defects.

Remineralization is a complex process that is influenced by the anatomy and physiology of the existing tooth structure, quantity and quality of saliva, the content and behavior of the bacteria biofilm, the presence or bioavailability of fluoride, and fluctuations of biofilm pH (2).

Most scientific evidence on therapeutic remineralization involves the use of fluoride, and numerous studies demonstrate the value of fluoride in the remineralization process (3). Fluoride alone as a remineralization strategy has not been shown to be sufficient to prevent dental caries at the population level, in addition to the controversy regarding whether this treatment improves the milky color of the porous enamel, or it just rehardens the surface layer with less effect on its appearance (4).

More recently, various forms of calcium phosphate have been included with fluoride in the therapeutic strategies. Tricalcium phosphate, amorphous calcium phosphate, and casein phospho-peptide-coated amorphous calcium phosphate have all been added to different oral care products. The scientific reports evaluating calcium phosphate remineralization strategies are mixed; some studies conclude there is a lack of evidence or no benefit, while others demonstrate improved results (5).

Since that hydroxyapatite (HA) is the main component of enamel, which gives an appearance of bright white and eliminates the diffuse reflectivity of light by closing the small pores of the enamel surface, (6) it is one of the most biocompatible and bioactive materials. It has a non-toxic, non-irritant, non-allergenic, non-mutagenic, non-carcinogenic property and anti-bacterial effect (7). In recent years, biomimetic approaches have been used to develop nano-materials for the reminerlization of early enamel lesions (8).

Compared to typical HA, nano-HA has some unique properties such as higher solubility, higher surface energy and optimal biocompatibility. Substituting bone and tooth with have several advantages including high toughness, high strength, high density, long shelf life and optimal biocompatibility (9).

In an attempt to combine the advantages of fluoride and synthetic nano-hydroxy apatite, fluoride ions were incorporated into the apatite lattice through precipitation and growth reactions. This substitution brings about a reduction in the volume of the unit cell, so that the chemical stability of the apatite lattice is greatly enhanced by the electrostatic bond between fluoride and the adjacent ions giving rise to a new form of nano-hydroxyapatite (10).

An adjunct method in the prevention of caries and treatment of initial defects is the use of laser it results in structural and chemical alterations of the enamel making the tooth more resistant to acidic challenge. Despite the abundance of research on caries inhibition effects of lasers, little data are available regarding their effects on the treatment of demineralized enamel (4).

This study is to evaluate the effect of combining laser irradiation and nano hydroxyapatite either in form of calcium hydroxyl apatite or in form of fluoride containing hydroxyl apatite on the surface properties of deminerlized enamel.

## Material and Methods

-Preparation of the experimental nanohydroxy apatite:

Two types of nano hydroxylapatite (nHAP); pure hydroxyapatite. (nHA) and Fluoro hydroxyapatite. (nFHA) were prepared by wet chemical precipitation method (11).

-Teeth samples preparation and grouping

Sixty extracted premolar teeth without visible caries or structural defects on enamel surface were collected, cleaned, and stored in artificial saliva solution at 37◦ C. The surface of each tooth was covered with an acid-resistant nail varnish, leaving a window of approximately 4×4 mm at the center of the buccal and lingual surface exposed. The crowns were separated from the roots, and the teeth were sectioned occlusogingivally through the center of the occlusal surface using a diamond disk. The tooth sections of the lingual surface were embedded in epoxy resin, and their surfaces were polished with sandpaper disks to be used in measuring the hardness, and the buucal sections were used for color assessment.

Each tooth was then individually immersed in 10 ml of a demineralizing solution. The demineralizing solution at pH4.8 consisted of 50 mM acetic acid, 2.2 mM CaCl2, and 2.2 mM NaH2PO4 for 2 weeks to create artificial white spot lesions. The teeth were sonicated for 10 minutes in 50% ethanol in order to remove any debris. Then, demineralized specimens were washed in distilled water for 10 minutes under stirring and then air dried.

The teeth were randomly allocated into two groups; Group 1: nHA and Group 2: nFHA. Each group is then subdivided into 2 subgroups (A and B) where two different in vitro remineralization procedures have been performed.

Group 1A (n=15); The first *in vitro* remineralization procedure utilizes a 10 wt% nHA aqueous slurries, slurries constituted of 20-60 nm sized nanocrystals. nHA nanocrystals aqueous slurries were applied for 10 minutes on the surfaces of the enamel at room temperature with 100% relative humidity and then removed by water washing and air dried.

Group 1B (n=15); Enamel windows were first exposed to irradiation from a fractional CO2 laser (wavelength 10.6 μm, 30 W, max. pulse: 80 ms, max.pilot laser power@630÷670:5mW. Classified by IEC 60825-1(2007-3; DEKA SmartXide, made in italy). The laser was operated in the dynamic mode with frequency of 100 Hz, 10 mJ of energy, and power of (4W), pulse duration 0.02 s and the beam was adjusted to cover a square area of 4×4 mm2 for 10 s per tooth. The laser’s handpiece was held manually at an approximate distance of 25 mm from the sample surface by one investigator and then were immediately treated with Nanohydroxy apatite. Vicker microhardness, color were then tested.

Group 2A (n=15); the first *in vitro* remineralization procedure utilizes 10 wt% nFHA aqueous slurries constituted of less than 100 nm sized nanocrystals. nFHA nanocrystals aqueous slurries were applied for 10 minutes on the surfaces of the enamel at room temperature with 100% relative humidity and then removed by water washing and air dried.

Group 2B (n=15); Enamel windows were first exposed to irradiation from a fractional CO2 laser with the same parameters as group 1 and then were immediately treated with nano Fluorohydroxy apatite, Vicker microhardness and color were then tested.

-Hardness testing

Microhardness was measured using a micro-Vickers hardness tester (Wilson hardness, model tukon 1102, weight 50 Kg, power supply Ac 100-240V, 47-63Hz) The Vickers microhardness tester has a diamond indenter with a square base and a pyramidal shape to allow penetration into samples. A weight is placed on top of the diamond indenter to force the tip into the surface of the enamel; the resulting impression is then measured using a high-power microscope with a scale to determine the diameter. The force from the weight and the diameter measurement are then put into an equation to calculate the hardness value. This hardness value serves as a Vickers Microhardness Number (VHN) that ranges between 100 and 1000.

The best point for load application was determined and a 200-g load was applied for 15 seconds to three points on the specimen surface, microhardness was then measured at the base line, after demineralization, after each remineralization procedure.

-Color examination

The specimens’ colors were measured at the base line, after demineralization, after each remineralization procedure using a portable Reflective spectrophotometer (X-Rite, model RM200QC, Neu-Isenburg, Germany). The aperture size was set to 4 mm and the specimens were exactly aligned with the device. A white background was selected and measurements were made according to the CIE L*a*b* color space relative to the CIE standard illuminant D65. The color changes (ΔE) of the specimens compared to baseline were evaluated using the following formula: ΔE CIELAB = (∆L*2 + ∆a*2 + ∆b*2) ½ Where: L* = lightness (0-100), a* = (change the color of the axis red/green) and b* = (color variation axis yellow/blue).

-Statistical Analysis

Data presented as mean and standard deviation (SD) values. Data explored for normality using Kolmogorov˗Smirnov and Shapiro-Wilk tests.

One˗way ANOVA was used to compare between different tested groups followed by Tukay’s post-hoc test for pair˗wise comparison when the omnibus ANOVA test is significant.

The significance level was set at *P* ≤ 0.05. Statistical analysis was performed with IBM® SPSS® (SPSS Inc., IBM Corporation, NY, and USA) Statistics Version 23 for Windows.

## Results

Microhardness and color results showed normal distribution. Post hoc comparisons indicated that that there was significant difference between tested groups’ (*P*≤0.001).

Results of microhardness testing showed as in ( Table 1); that demineralization resulted in significant reduction on mean Hardness (220.15±2.77 VHN) compared to Baseline (283.97±10.83 VHN, there was a significant increase of microhardness of the enamel surface after being treated with nano hydroxyapatite whether the pure type(293.6±5.97 VHN) or the flouro apatite (279.81±8.36 VHN) one, laser application followed by nano hydroxyapatite application showed the highest mean hardness ; nHA (341.57±5.99 VHN), nFHA(320.67±3.09 VHN).

**Table 1:**
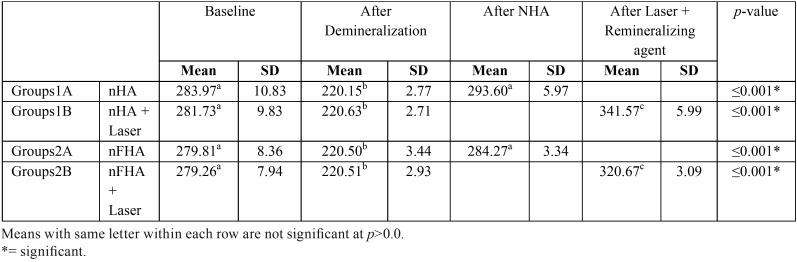
Mean and SD for Hardness for different tested groups of NHA and FHA.

The pure type of nanohydroxyapatite (nHA) showed the highest significant mean Hardness values with and without laser application compared to nano flurohydroxyapatite type (nFHA) as showed in ( Table 2).

**Table 2:**
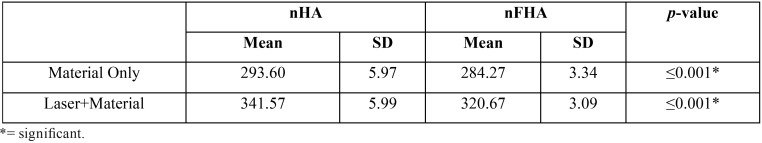
Mean and SD for Hardness for the effect of different remineralizing materials.

The color difference between the baseline and demineralization stages (ΔET1–T2) was greater than 3.3 units in the two groups (nHA, nFHA ). After nanohydroxyapatite application the color difference between the baseline and remineralization stages (ΔET1–T3) was less than 3.3 units in the two groups. The LASER treated groups showed significantly the lowest value of the color difference between the baseline and remineralization stages (ΔET1–T4) which was less than 3.3 units in the two groups as showed in ( Table 3).

**Table 3:**
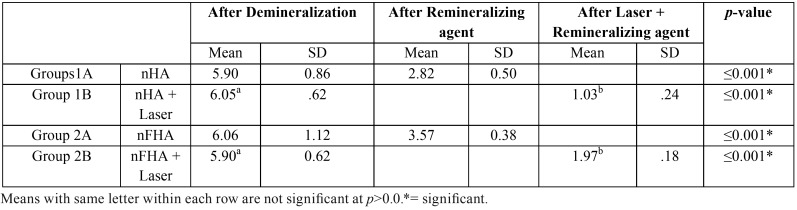
Mean and SD for ΔE for different tested groups for Demineralization-Remineralization protocol.

The pure type of nanohydroxyapatite showed the lowest significant mean ΔE values with and without laser application compared to nFHA as showed in ( Table 4).

**Table 4:**
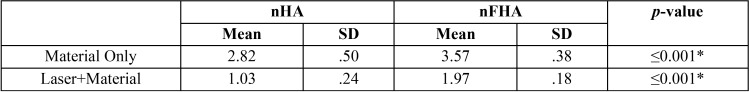
Mean and SD for ΔE for the effect of different remineralizing materials.

## Discussion

In this study microhardness is used as an indication for testing remineralization as many reports indicate that tests of surface microhardness provide a sensitive measure of mineral loss. This experiment used a Vickers Microhardness Tester to measure vicker hardnes value (VHN) values of tooth enamel.

Results of microhardness testing showed that Demineralization resulted in significant reduction on mean Hardness (220.15±2.77 VHN) compared to Baseline (283.97±10.83 VHN), which might be contributed to enamel weakness by increased pore size due to mineral removed from the enamel surfaces as a result of dissolution of hydroxyapatite. There was a significant increase of microhardness of the enamel surface after being treated with nano hydroxyapatite whether the pure type (293.6±5.97 VHN) or the flouro apatite (279.81±8.36 VHN) one, indicating that nHAP induces a consistent enamel remineralization by forming a homogenous apatite layer on the demineralized surfaces of enamel after treatment, which can be explained by the facts of ; NHA has hydrophilic and wetting characteristics and is capable of producing a thin but tightly bound layer on the tooth surface, resulting in higher surface hardness and remineralization (7). Besides, nano-hydroxyapatite also acts as filler because it repairs small holes and depressions on enamel surface, a function enhanced by the small size of the particles that compose it (6). In addition, apatite formed in an environment containing more calcium and phosphate is expected to contain less CO2 and less Mg by replacing them with calcium and phosphate, making enamel more acid resistant and increasing its surface hardness levels (12). This result is in agreement with Swarup JS *et al.* (1), Rathi N *et al.* (13), and Ebadifar A *et al.* (7).

Laser application followed by nano hydroxyapatite application showed the highest mean microhardness; nHA (341.57±5.99 VHN), nFHA (320.67±3.09 VHN). The increased microhardness of the softened enamel following laser irradiation has been attributed to the ultrastructural changes including crystal size growth and recrystallization of porous enamel as a result of high temperature rise in the surface (14). It is possible that the chemical and structural alterations facilitated nanohydroxyapatite deposition, thus improving microhardness of enamel surface (15).

The pure type of nanohydroxyapatite (nHA) showed the highest significant mean Hardness values with and without laser application compared to nano flurohydroxyapatite type (nFHA) which could be due to the ability of nHA to fit more into the prismatic and interprismatic enamel structure creating a more homogenous surface, on contrary of nFHA which due to either the shape or size of the prepared nanoparticles might have blocked any further transport into deeper lesions parts by decreasing the pore volume of the surface layer and obstructing the diffusion pathway, and this could have inhibited further remineralization.

In spite of the fact that color matching is routinely performed using a visual method, in the present study, color differences or efficacy due to the whitening effect of nanohydroxy apatite, were quantified with a spectrophotometer.

It is believed that a ΔE (discrepancy between two hues) exceeding 3.3 units indicates color mismatching, as it would be clinically visible in any site by independent observer (15).

The color difference between the baseline and demineralization stages (ΔET1–T2) was greater than 3.3 units in the two groups (nHA, nFHA ), indicating the creation of clinically visible white spot lesions. The white appearance is due to an optical phenomenon that is caused by mineral loss in the surface or subsurface enamel (16). Enamel crystal dissolution begins with subsurface demineralization, creating pores between the enamel rods. The resulting alteration of the refractive index in the affected area is a consequence of both surface roughness and loss of surface shine and of alterations in internal reflection, all resulting in greater visual enamel opacity, as porous enamel scatters more light than sound enamel (17).

After nanohydroxyapatite application the color difference between the baseline and remineralization stages (ΔET1–T3) was less than 3.3 units in the two groups, implying that the enamel appearance was not different from the intact enamel surface when evaluated by independent observers, which can be attributed to the ability of nanohydroxy apatite to adhere to the tooth surface without affecting or chemically altering the deeper tooth tissue. The hydroxyapatite material itself is white, and it is assumed to reflect more light when it is applied as a thin surface layer than the more transparent natural enamel, because this transparency usually means a loss of reflected light due to transmission or scattering to directions other than the observer’s eye (18), in addition to nano-hydroxyapatite ability of being deposited in the cavities and defects of enamel surface enhance smoothness. As the particle of nano-hydoxyapatite is fairly small sized, it can enter into the enamel surface continuously and fill the vacant position of enamel crystal. Although it is very dense, partial penetration of certain ions and molecules through the enamel structure is possible because it contains small and intercrystalline spaces, rod sheaths, enamel cracks, and other defects (19).

The LASER treated groups showed significantly the lowest value of the color difference between the baseline and remineralization stages (ΔET1–T4) which was less than 3.3 units in the two groups, and this was possibly due to creation of microscopic pores within the structure of laser irradiated which enhanced more nanohydroxyapatite particles entrapment and deposition on the enamel surface improving the color.

The pure type of nanohydroxyapatite showed the lowest significant mean ΔE values with and without laser application compared to nFHA which might be due to more precipitation of fluoride on the enamel surface that induced a negative effect on the color of demineralized enamel.

Furthermore, several individual factors could have potential impact on remineralization (e.g., behavioural changes, activity of the lesion, depth of the lesion, diet, stimulation of salivary flow, antibacterial and plaque removal strategies, brushing with fluoride toothpaste) and these factors may modulate the natural process of lesion arrest (or repair) (2).

## Conclusions

The nano-hydroxyapatite is a revolutionary material with a wide use in dentistry. With regard to restorative and preventive fields, nano-hydroxyapatite has remarkable remineralizing effects on initial lesions of enamel, certainly higher when combined with laser application.

1- Application of fractional Co2 LASER before nano-hydroxyapatite caused significant increase in the surface microhardness of enamel and was more effective in restoring the teeth color than using nano-hydroxyapatite alone.

2- Pure type of nanohydroxyapatite gave better surface microhardness and color results than the nanofluroapatite type.
